# Abstract of Meteorological Observations for August and for the Summer of 1856, Made in the Twenty-fourth Ward, Philadelphia

**Published:** 1856-10

**Authors:** James A. Kirkpatrick


					﻿R	Barometer. Thermometer.	a>
Reduced to 32°._________ St
^2	1856.	I Solar	Remarks.
•	Ausust.	„ ., Mcan	Mean Ex- Radi- Rel. Forceof	Dew	„	...	J3	aS
© .©	,	°	Daily Daily	Daily Daily treme ation. Humid. Vapor	Point	Rain Prevailing	©	-r
'*•	Mean Range. Mean Range Range 2P.M 2 P.M. 2 P.M. 2P.M.	Winds.	a
j '■§"<	Inches. Inches. Deg. Deg. Deg. Deg. Hunds. Inches. Deg. Inches. Points.	§	Jq "S
11 i BSSSHt i! Sit (tat	i -!i
6	29.577 '.056 74.3 lo 19	42	41	«7 55'2 S NW. Cloudy; rain morning and afternoon.	^7-7, ■	?
7	29.567 .022 76.8 2.2	25	10	44	. 531 60.7	(Var.) an.d aft dear; ev. cloudy, with ram, thunder and lightning, fs
£	8	29-508 -°59 75.2	5.3	16| 5J	65	.723 69.6	i*692	<Var-)	Cloudy ; morning light showers; 4|	until 7, P.M. heavy rain, 2
9	29.590.082 71.3 6.5	19	25	53	.491 58.5	W.	^Hh ZiatZ for a few minutes.	g	£?©
11	29.’53? .091 W 4i3	I1?	53	'.71169.1	0.015 SSW.	KX“Bmom^SClear; aftern°°n C1°Udy’	S	"3?
.S^	If	291-8 060 HI	It H	-03® 60-0	0-072	WSW	o5oSH7toTf.M“ r«iWs^.	5-3
•2	14	29’490 '023 -50 1*7	90	34 a- ’-a? fif’I	(Var) Cloudy ! evening, distant thunder and lightning.	j ® ©
15	“ :^7	Il	it	&	Morning and aft. cloudy; evening clear.	-
17	SISK. 11	21	io2	11	.401	gt	Clear; evening, Zoning in North.	£	*’S>
19	29.391 yS:?’!?	10	17	ft	1?1	®	1-165 Jv”:	c\oud^ racing?a^Oday^6^ ’	2	*
g Hl^tl 1?	£	«1	0,008 (Var)	ev.elear. Baxter lowest 29.155. £	2 S
■3|3	H 29540 ^7^3 93	9? 9R Tt 'tot tot	NW> Cloudy, a few drops of rain about noon.	N 03
23	2916 ’.^9 7W 21	29	tl 571 62 8	SW.’ Morning cloudy; afternoon and evening clear.	5
P G .	ot 29 497 058 64*7 R3 1- il to ’at- -t’r	NW. Morning cloudy; afternoon and evening clear.	o q S ,t3
© g	“* *	’	64.7 8.3	15	16	69	.47a o7.6	M.. cloudy; 1|, P.M. high wind and a few drops of rain; wind <d »o 03 "g
•2	on ?Q7fil Ottd -On K rr	™	storm from the North, continued about 20 minutes.	£p m
O JQ	T 29 833 ’n79 ftc'o H OQ oo ?!	’^o2 !o’n	V' Clear; night very heavy dew. T/ierTnom^er ZowesU7°.	§ S
28	29 754	080	70 5	52	93	191	a«	’a?q	t«n	cp	Clear; morning first hoarfrost observed.	Bar. highest 29.846.	PS	2	®	g
.	oo 2Q’ooq 70 7 o’o ?!	1?‘a	4!	‘449	• Morning and after noon cloudy; evening clear.	>> g o m
29	*29*632	107	63 0	0’7	iq	-i?	12	to"?	0-195	SW-	M. and aft. rain showers; between 3 and	4, P.M. tli’r. and It’ng.	3	fl	3	°
u?	90?S	iw	««q	«l	I!	IS	18	1358	194.	NW*	Morning and evening clear; aft. cloudy.	“	g	“
■q ©	11	'	5.3	28	37	47	.464 56.9	SW. Morning and afternoon cloudy; evening clear.	o 2 os tq
£ © Cfl Means for Aug. 29,565 .092 72.2 4.1	29	24}	56	.545 60.8	5.186 S. 89° 15'W. 25-100.	© .
“Summer.	.093 76.5 4.2	53 I 52	-558 61.4	7.990 S. 68° 33'W. 36-100.	S H >
~-----------------------------------------------------------------------------------------------------------------------------------------a g
				

